# Impact of a Traditional Dietary Supplement with Coconut Milk and Soya Milk on the Lipid Profile in Normal Free Living Subjects

**DOI:** 10.1155/2013/481068

**Published:** 2013-10-24

**Authors:** R. A. I. Ekanayaka, N. K. Ekanayaka, B. Perera, P. G. S. M. De Silva

**Affiliations:** ^1^Institute of Cardiology, National Hospital of Sri Lanka, Colombo 8, Sri Lanka; ^2^Medical Research Institute, Colombo 8, Sri Lanka; ^3^Faculty of Medicine, University of Ruhuna, Galle, Sri Lanka

## Abstract

*Background*. The effects of coconut fat and soya fat on serum lipids are controversial. This study was designed to investigate the lipid effects of coconut milk and soya milk supplementation on the lipid profile of free living healthy subjects. *Methods.* Sixty (60) healthy volunteers aged 18–57 years were given coconut milk porridge (CMP) for 5 days of the week for 8 weeks, followed by a 2-week washout period, subsequent to which they received isoenergetic soya milk porridge (SMP) for 8 weeks. *Results*. The LDL (low density lipoprotein) levels decreased with CMP and reached statistical significance in the total study population and in the >130 baseline LDL group. The HDL (high density lipoprotein) levels rose significantly with CMP supplementation (*P* = 0.000). *Conclusions*. We conclude that coconut fat in the form of CM does not cause a detrimental effect on the lipid profile in the general population and in fact is beneficial due to the decrease in LDL and rise in HDL. SMP will be of benefit only in those whose baseline LDL levels are elevated.

## 1. Introduction

Abnormal blood lipids are generally accepted as a major risk factor for ischaemic heart disease amongst all ethnic populations [1]. However, dietary modifications which would help prevent ischaemic heart disease remain controversial as data from different studies are at times inconsistent [2–8]. It must be emphasized that test diet results cannot always be confirmed in community studies when usual day-to-day diets are consumed which are often quite different from test diets.

The above statement is true for coconut and coconut based foods, which, being a relatively cheaper fat, is consumed extensively in Asia mainly due to age-old sociocultural culinary practices. The fact that coconut is cultivated locally also contributes in no small measure to its integration into the daily diet of Asians. However, being primarily a source of a saturated fat, coconut has been incriminated as a risk factor for the high incidence of atherosclerotic disease seen in Asians.

Early studies regarding the role of coconut in atherogenesis were performed on animals such as rabbits, mice, rats, gerbils, dogs, and monkeys where hydrogenated coconut fat was used in the test diet. This has prompted a significant debate regarding the relevance of these animal studies in humans [9, 10]. Some have gone to the extent of even recommending coconut as a dietary item which would help reduce or prevent coronary arterial disease on the basis of the “Polynesian paradox,” where it is claimed that the high fat intake in the form of coconut has provided some protection against atherosclerosis amongst the Polynesian islanders [6].

Many meta-analyses published in recent years which have attempted to evaluate the relationship between dietary intake and resultant morbidity from ischaemic heart disease have raised doubts regarding the role played by saturated fats in atherosclerotic disease.

In 2004, Mozaffarian et al. published a study on atherosclerosis amongst postmenopausal women [11]. The aim of the study was to find a relationship between macronutrient intake and atherosclerotic disease as determined by coronary angiography. The mean total fat intake was 25 ± 6% of energy. An unexpected finding of their study was that a higher saturated fat intake led to less coronary disease as indicated by a lesser mean coronary diameter and lesser progression of stenosis. Surprisingly, PUFA intake was associated with greater progression when replacing other fats.

Mente et al. in 2009 [12] published a meta-analysis attempting to elucidate the causal link between dietary fat intake and coronary heart disease. They found strong evidence for a valid beneficial association between intake of vegetables, nuts and a Mediterranean dietary pattern and harmful association with trans fat containing foods and high glycaemic index/load foods. There was insufficient evidence to establish causal links between intake of polyunsaturated fat, total fat, alpha linolenic acid, eggs, and milk and ischaemic heart disease.

In yet another meta-analysis of prospective epidemiologic studies by Siri-Tarino et al. in 2010 [13] which included 347,747 subjects with a follow-up period ranging from 5 to 23 years the authors concluded that there was no significant evidence to link increased saturated fat intake with enhanced risk for ischaemic heart disease.

A WHO [14] report in 2003 concluded that there was no evidence to directly link the quantity of daily fat intake to increased risk of coronary vascular disease. In, 2012, a standard text book on cardiology commented that the evidence for the effect of saturated fat on CVD risk factors or end points such as blood pressure or stroke is limited [15].

Clearly the question of dietary intervention for prevention of atherosclerosis is not yet definitively solved. Many studies provide acceptable evidence to establish an inverse relationship between PUFA intake and coronary vascular disease [16–18]. Reducing saturated fat too is shown in well-conducted studies to reduce risk of ischaemic heart disease [19, 20]. However, as stated before the evidence is not universally confirmatory of these statements.

Further work in this field is needed because normalizing a lipid profile is not the health care providers' primary target. It is reducing the burden of ischaemic heart disease that the clinicians and dieticians would consider as their foremost aim. Therefore, dietary studies are required which have a strong bias towards clinical outcomes which also investigate the effects of complex food items which are consumed by the population in day-to-day life.

Coconut is consumed in three main forms in Asian countries. The grated kernel is used mainly in the preparation of “salad” of the “sambol” type. Coconut milk is used extensively in preparation of curries, whereas coconut oil is used for frying and so forth. For this study, coconut milk was the chosen modality of coconut consumption as the study design was one of the supplementations of the usual diet by the test fat and not a substitution. It was thought that coconut milk would be a suitable form to study, firstly because preparations of coconut milk were available of constant nutritional content and secondly because a common supplementary beverage used by many Sri Lankans particularly in the villages could be prepared using coconut milk or soya milk as the base. The soya also being available in milk form with constant nutritional content, the porridge could be prepared in the same manner to obtain a set of data for comparison. 

It was recognized as being very important to ensure that the soya and coconut used should be in the natural form as much as possible, without modification by hydrogenation or use of production methods promoting the formation of trans fatty acids. The aim was to study the effects of “natural” coconut milk and soya milk supplementation in “healthy” free living Sri Lankans who were consuming a customary “usual” Sri Lankan diet.

## 2. Methods

### 2.1. Subjects

Participants (*N* = 60) were volunteers working in the National Hospital of Sri Lanka, Colombo, and Medical Research Institute, Colombo. A local advertisement was used to recruit the participants. As the study required that the enrolled subjects should consume freshly prepared porridge at least 5 days of the week, employees and students physically present in the two study centers were selected as subjects on first-come basis, purely for the ease of supervised administration. All subjects selected were healthy, in that they had no physical complaints and were not on any medications. A dietary questionnaire confirmed that they had no dietary habits which could be considered out of the ordinary when compared to a usual Sri Lankan diet.

This study was ethically approved by the ethics committee of Medical Research Institute, Colombo 8, Sri Lanka.

### 2.2. Study Design

There were two study feeding periods, each of 8-week duration. As it takes several weeks for plasma lipids to equilibrate with tissue lipids [21, 22], a period of 8 weeks was selected as the feeding time, as had been done in previous studies [23]. 200 mL of coconut porridge was offered for the first 8 weeks, followed by 2-week washout period. Subsequently, 200 mL of soya porridge was offered for 8 weeks. Serum lipids were estimated at (i) baseline, (ii) end of the coconut milk porridge (CMP) supplementation, (iii) end of the washout period, and (iv) end of the soya milk porridge (SMP) supplementation. Thus each subject who completed the study had four assays of serum lipids. 

The subjects were advised to continue on their customary diet. Consent was obtained to consume an entire glass of porridge provided for at least 5 days of the week. 

### 2.3. Dietary Protocol

The CMP was prepared using commercially available coconut milk (CM) in powder form. The nutritional content of the CM powder used is given in Table 1.

The porridge was prepared to contain ~1075 kJ (260 kcal) (which is approximately 10% of the calorie requirement of a physically active male).

The soya milk (SM) porridge was prepared using commercially available SM powder which had the nutritional content given in Table 1. To increase the palatability, 200 g of sago was added for every 50 patients, which gave 4 g of sago per person (providing negligible contribution to the nutritional content).

A 12-hour fast was mandatory before blood was sampled. The lipid profiles were performed in a single laboratory by a single technician. In order to confirm that the test results were reliable and reproducible, 20% of the serum samples were sent to another laboratory where a single technician performed a repeat assay of serum lipids as an intrastudy crosscheck. 

The lipid assays were done using reagents supplied by AMP Diagnostic (Austria) and Diagnostic Systems International (Germany). 

### 2.4. Statistical Analysis

The data were analyzed using SPSS 17 for Windows (SPSS, Inc., Chicago, IL, USA). Data are presented as means with their standard deviations and assessed for normality using Kolmogorov-Smirnow test. Paired Student's *t*-test was used to evaluate within-group treatment effects. Generalized estimating equations were used to assess the effects of the intervention on the lipid profile by including baseline values of the parameters as covariates. Pearson's correlation analyses were performed to identify the change of treatment effect with the respective baseline parameter. An *α* level of *P* < 0.05 was considered statistically significant.

## 3. Results

Sixty healthy individuals were recruited for the study. The participants were aged 18–57 years, with a mean age of 42.6 years (SD = 10.3). Half of the participants (*n* = 31) were males. The average body mass index (BMI) was 19.7 kg/m^2^ (SD = 3.3) for males and 19.2 kg/m^2^ (SD = 2.8) for females. All female participants were premenopausal.

Table 2 presents the mean, standard deviation, median, and range of the pre- and postsupplementation data sets of the response variables (i.e., HDL and LDL).

The difference in the mean values between the post and the base HDL levels with the CMP supplementation was 9.6 mg/dL (standard error of the difference between the means (SEM) 1.6 mg/dL), and the difference was statistically significant (*P* < 0.01). The difference in the mean values between the post and the base LDL levels with the CMP supplementation was –14.9 mg/dL (standard error of the difference between the means (SEM) 6.2 mg/dL), and the difference was statistically significant (*P* = 0.02). Therefore, CMP supplementation is effective in increasing the HDL and decreasing the LDL levels of the study participants. 

The difference in the mean values between the post and the base HDL levels with the SMP supplementation was 1.8 mg/dL (standard error of the difference between the means (SEM) 1.3 mg/dL), and the difference was not statistically significant. The difference in the mean values between the post and the base LDL levels with the SMP supplementation was –11.8 mg/dL (standard error of the difference between the means (SEM) 6.7 mg/dL) and indicated a marginally significant difference (*P* = 0.09). Therefore, SMP supplementation seems to be effective in decreasing the LDL levels but has no significant effect on the HDL levels.

When compared with respective baseline values, the changes observed in HDL and LDL after the CMP supplementation were found to have no correlation: HDL (*r* = −0.14, *P* = 0.29) and LDL (*r* = −0.17, *P* = 0.19). The changes observed in HDL and LDL after the SMP supplementation were found to be negatively correlated with their respective baseline values for HDL (*r* = −0.35, *P* < 0.01) and LDL (*r* = −0.46, *P* < 0.01), raising the possibility of regression to mean effect in data.

Using generalized estimating equations (GEE), the data was analyzed to see whether the responses to CMP and SMP are dependent on baseline values of HDL and LDL. It was observed that the type of supplement (*P* < 0.01) and baseline HDL level (*P* < 0.01) significantly predict the postsupplementation HDL levels. CMP supplementation is more effective in increasing the level of HDL compared to SMP supplementation. However, no significant difference was found between CMP and SMP supplementation in reducing LDL levels. Only baseline LDL values (*P* < 0.01) significantly predict the postsupplementation LDL values.

Therefore, we categorized the subjects based on their baseline values of the parameters consedring the currently accepted therapeutic targets. Hence HDL > 40.0 mg/dL and LDL < 130.0 mg/dL were used as cutoff values [24], and the effects on the study parameters were compared (Table 3).

CMP supplementation had an effect of increasing HDL levels, irrespective of the baseline HDL level, but no such effect was observed with SMP supplementation. When the baseline level of LDL was <130.0 mg/dL, no significant difference in the mean values between the postsupplementation and base LDL was found in both CMP and SMP supplementation, although there was a clear trend towards lower LDL level with the CMP supplementation. When the baseline level of LDL was >130.0 mg/dL, both CMP and SMP supplementation showed a significant reduction in the mean LDL levels.

**Figure 1:**
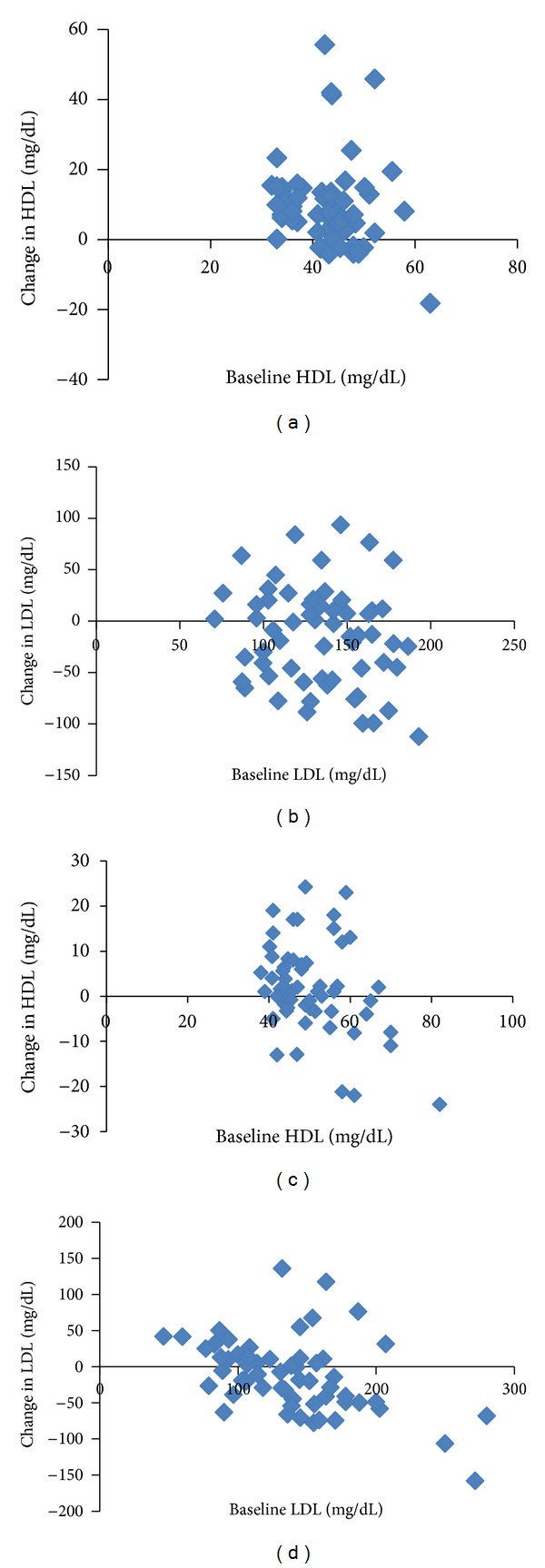
Correlation between baseline HDL, baseline LDL, and changes observed in HDL and LDL. (a) Baseline HDL and changes in HDL (CMP) (*r* = − 0.14, *P* = 0.29), (b) baseline LDL and changes in LDL (CMP) (*r* = − 0.17, *P* = 0.19), (c) baseline HDL and changes in HDL (SMP) (*r* = − 0.35, *P* = 0.006), and (d)  baseline LDL and changes in LDL (SMP) (*r* = − 0.46, *P* = 0.001).

Figure 1 demonstrates correlations between baseline LDL and HDL levels and changes effected by CMP and SMP supplementation.

**Figure 2:**
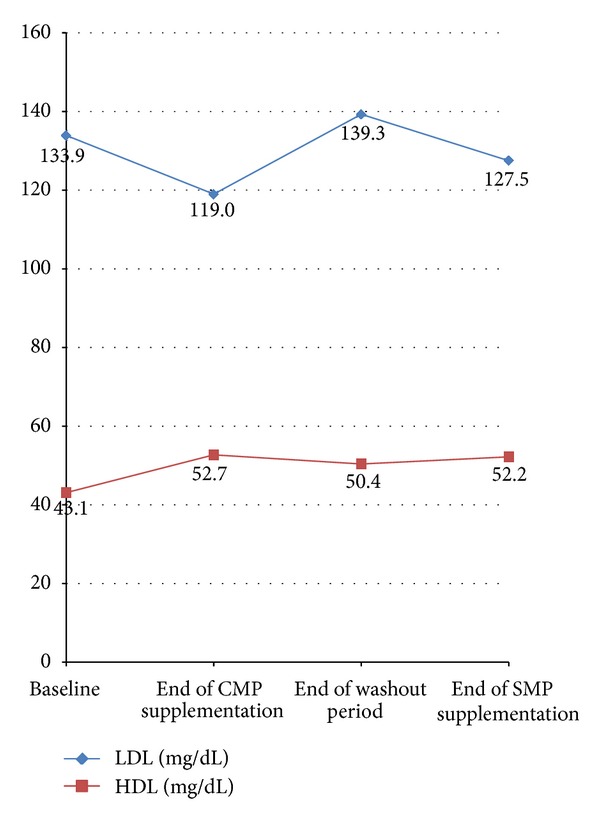
Graphic representation of LDL and HDL changes following supplementation with Coconut milk and Soya milk porridge.

Figure 2 summarizes the LDL and HDL changes which occurred with CMP and SMP supplementation.

## 4. Discussion 

### 4.1. Effect of Coconut Milk Supplementation on the LDL Levels

An unexpected finding in our study was the statistically significant decrease in LDL level following coconut milk supplementation.

Coconut fat, consisting mainly of saturated fatty acids, is commonly held to contribute towards a rise in the LDL. The early metabolic ward studies of Keys [25] indicated that the adverse effects of saturated fats on total cholesterol were twice the benefit accrued from polyunsaturated fatty acids. Trials such as MRFIT (Multiple Risk Factor Intervention Trial) [26], CPPT (Coronary Primary Prevention Trial) [27], and DASH (Dietary Approaches to Stop Hypertension) [28] indicated the benefit of reducing saturated fat in the diet. Hence, standard dietary guidelines such as the ATP diet recommend lowering of saturated fatty acids to less than 10% in step I and less than 7% in step II. ATP III TLC programme suggests that a 1% reduction in saturated fatty acids would reduce LDL by 7%. 

The formulae developed by Keys and Hegsted take as a premise that saturated fatty acids shorter than 12C atoms have no appreciable effect on total cholesterol [29, 30]. The major saturated fatty acids in coconut fat are lauric (C12), myristic (C14), and palmitic (C15) which together give 72.2% of the total saturated fatty acids in coconut (Table 4).

All saturated fatty acids are not the same where lipidogenicity is concerned. Long chain fatty acids are well established as being lipidogenic, whereas medium chain saturated fatty acids are thought to be neutral in this aspect. Coconut fat has only 36.5% (per 100 g of total fat) of fatty acids which could be classified as long chain. 45.8% consists of lauric acid which has a 12C chain. Controversy exists as to the classification of lauric acid as either a long chain or a medium chain fatty acid. Some lipidologists classify it as a medium chain fatty acid solely based on its chain length [31–33], whereas other workers prefer to classify it as a long chain fatty acid basing their conclusion on its metabolic effects [34]. If the latter conclusion is accepted, approximately 80% of the saturated fatty acids of coconut would consist of long chain fatty acids which should contribute to a significant rise in LDL levels. 

Not all investigators agree that coconut fat raises the LDL. Reiser in 1973 [35] argued that coconut fat plays no part in raising total cholesterol. His meticulous analysis of the studies done up to that date casts serious doubts on validity of the statement that coconut fat contributes to hypercholesterolaemia. In this context, the view of certain lipidologists that lauric acid (12C) is a medium chain fatty acid gains ground as coconut fat having a high proportion of medium chain fatty acids would be metabolically neutral. The study of Cater et al. [33] disagreed with this view and concluded that medium chain fatty acids have one half the potency of palmitic acid in increasing LDL. 

Another factor which must be considered in this debate is the amount of coconut fat which was used in various studies. An indicator that a very heavy load of coconut fat may in fact give rise to a different cholesterolaemic response compared to a lesser load comes from the study of Pukapuka and Tokelau islanders of the Polynesia. The Tokelau islanders derive 63% of their energy from coconut and have serum cholesterol levels which are 30 to 40% higher than those of the Pukapuka islanders, in whom coconut accounts only for 34% of total energy intake [6]. In the Pukapuka community the average serum cholesterol is in the acceptable range of 148.9 to 194.2 mg for all age groups. 

The metabolic status of lauric acid may be reflected in the deposition pattern of fatty acids in the fat biopsies of the Pukapuka and Tokelau islanders. The deposition of lauric acid is found to be equal in both populations, although one had a coconut fat consumption which was approximately twice that of the other. The major constituent in the fat deposits was long chain fatty acids, which are noted to be atherogenic. This finding suggests that lauric acid may not be important in lipid deposition in the human body. 

The trials which demonstrated an adverse effect on the lipid profile utilized coconut fat either as oil or solidified by hydrogenation. In either case it was the extracted coconut fat that was used. However, although not conclusive and not universally accepted, some evidence does seem to favor the conclusion that saturated fat from coconut does in fact increase the LDL. The hypothesis that saturated fatty acids raise the LDL cannot be easily disregarded as the proposed mechanism by which the LDL is raised, that is, reduction of LDL receptor density, has been adduced and supported by many pieces of experimental evidence. 

The LDL receptor appears to be vital in controlling the LDL levels [36, 37]. In general, lower the number or one less the efficiency of the LDL receptors, higher will be the plasma LDL level. LDL receptors are present on surfaces of most mammalian cells but are primarily expressed in the liver, adrenal cortex, and ovarian corpus luteum. Because the LDL receptor is under feedback control, it is possible to increase the population of LDL receptors by depriving cells of cholesterol content. Goldstein and Brown [38] pose the crucial question, “how a high intake of exogenous saturated fats and cholesterol lead to an increase in plasma LDL, an endogenous lipoprotein that originates in the liver and not in the intestine?” The limited number of receptors being unable to cope with the excess influx of lipids to the liver and the reduction of the LDL receptors by the excess hepatic cholesterol are two facts which have been recruited to answer this problem. With excessive dietary saturated fats and cholesterol, the liver produces a greater load of VLDL and LDL, and because the removal capacity by the receptors is limited the serum LDL levels rise. In addition, the elevated hepatic cholesterol content suppresses the expression of LDL receptors. Thus the LDL rises significantly with pure saturated fat. However, the downregulation of the LDL receptor population by saturated fat has been queried in some animal experiments, where the expected reduction in the receptor mRNA level could not be demonstrated when a saturated fat load was included in the diet [39, 40].

Even if it is accepted that coconut fat consists of medium chain saturated fatty acids, the effect on the LDL should be neutral, and it is not possible to predict or explain a reduction in the LDL on this basis. Hence the reduction in LDL which was demonstrated in our study cannot be explained on the fatty acid content of the coconut milk preparation used.

The studies from the Polynesian islanders differ from metabolic ward trial studies as the Polynesians use natural coconut in its entirety consuming both kernel and milk and not only isolated saturated fat. Hence the possibility arises that other constituents of natural coconut could reduce LDL, while its fatty acid content remains largely neutral on lipids.

Trinidad et al. [41] studied the effect of coconut flakes on the serum cholesterol levels of healthy individuals with moderately raised cholesterol levels of 259 to 283 mg. The test foods included coconut flakes which provided 15% and 25% dietary fiber from coconut. The results showed a significant reduction in the total cholesterol and LDL with both 15% and 25% fiber containing coconut flakes. Thus the fiber content which included both soluble and insoluble forms could have contributed towards the cholesterol reduction as concluded by the authors. Coconut milk used in our study would contain mainly the soluble fiber which is thought primarily to be beneficial in terms of lipid reduction. The US Department of Agriculture estimates that a cup of raw coconut milk would contain 5.3 g of fiber. Considering the ATP III guidelines for daily fiber intake which is 20–30 g, our study population would have received 25% of their daily recommended intake in addition to the relatively high fiber content of the average Sri Lankan diet which is 22.9 ± 35.0 g [42].

Padmakumaran Nair et al. [43] have suggested that the protein content of coconut may have an effect in reducing LDL. When healthy human volunteers were fed coconut kernel along with the oil, the effect on the lipid profiles was beneficial compared to feeding coconut oil alone. That the kernel protein may play a part has been elegantly demonstrated in a rat model, where the same phenomenon is seen. The kernel protein has a very low lysine/arginine ratio (2.13/24.5), which may explain the metabolic effect seen in the rat model, that is, (i) increased degradation of cholesterol in the liver, (ii) decreased esterification of cholesterol, and (iii) decreased intestinal synthesis of cholesterol. These effects finally counteract the increased hepatic cholesterol synthesis also found. Coconut kernel protein is not unique in this effect. Many plant proteins probably possess the property of LDL reduction. In our study the coconut milk supplied 2.44 g of protein daily which could have also contributed to the beneficial effect on LDL.

Thus there seems to be experimental evidence to support the view that the lipid effects of coconut milk/kernel would be different from those of the pure oil due to the salutary effects of the fiber and protein. Hence coconut milk and coconut kernel would reduce LDL cholesterol, whereas the oil itself could be neutral, leading to a net reduction in LDL.

An important finding in our study is that the LDL levels came back to baseline 2 weeks after the coconut milk supplementation was terminated. This significant finding is a strong indicator that the coconut milk supplementation itself was responsible for the reduction in the LDL in the first place. 

### 4.2. Effect of Coconut Milk Supplementation on the HDL Level

In our study the HDL levels showed a statistically significant increase following the coconut milk supplementation.

A rise in HDL while ingesting a saturated fat rich diet is well documented in human subjects [44–46]. This effect appears at first glance to be paradoxical in terms of the epidemiology of ischemic heart disease as saturated fats are generally thought to be atherogenic, while their HDL lowering action is deemed to be protective against atherosclerotic vascular disease. This discrepancy highlights the necessity to rethink and reevaluate the lipid effects of various fats with special emphasis on clinical outcomes, for the latter should be the decisive criterion for prescribing a diet for which biochemical reasons could be adduced both for and against.

The specific saturated fatty acids which have the most HDL raising effect have not been identified, and there is a paucity of published studies on this aspect. The present study provides evidence that saturated fat derived from coconut is efficacious in raising HDL levels.

Mensink and Katan [47] performed a meta-analysis of selected trials to determine the effect of fat type on the total cholesterol and HDL levels. They found that the effect of dietary fat on the total cholesterol/HDL ratio differed markedly from the effect on the LDL level. If the saturated fat was replaced by carbohydrate, the HDL levels remained unchanged. If, however, *cis*-polyunsaturated fatty acids replaced the saturated fat, the total cholesterol/HDL ratio decreased indicating significant rise in the HDL fraction. When the effect of specific fatty acids was investigated, it was found that lauric acid had the most profound effect in increasing the HDL level. It was further demonstrated that lauric acid decreased the total cholesterol/HDL relative to carbohydrate, indicating that it raised HDL more than it raised LDL cholesterol. In our study the CM beverage would have provided a significant load of lauric acid to the daily diet of the study subjects.

The estimated saturated fat content in the normal diet would be 52 g, which would have increased to 75 g by the addition of the coconut milk beverage. The contribution of saturated fat to the daily caloric intake would be approximately 20%, which would be higher than the recommended <10%. Although considerable concern arises regarding ingesting this amount of saturated fat intake, it must be emphasized that the final blood lipid levels were not adversely affected by this quantity of saturated fat and in fact showed a definite change for the better. We postulate that this is partly due to the other components of the food item which contained the saturated fat.

### 4.3. Effect of Soya Milk Supplementation on the LDL Levels

The reduction in the LDL levels seen in our study following the soya milk beverage supplementation is to be expected as evidenced by the extensive literature reporting that increased intake of polyunsaturated fatty acids leads to a reduction in total cholesterol and LDL levels. Of the n-6 polyunsaturates, linoleic and oleic acids are thought to be the important fatty acids in reducing LDL.

Soya oil is rich in linoleic acid and oleic acid. The soya milk beverage used in our study would have provided approximately 7.9 g of linoleic acid, 3.4 g of oleic acid, and 1.3 g of linolenic acid, which gives a total of 12.6 g of polyunsaturated fatty acids, representing a doubling of the normal polyunsaturated fatty acid content in the average diet of the study subjects.

Many metabolic processes have been suggested via which the polyunsaturated fatty acids could reduce the LDL levels. The n-6 polyunsaturates have been demonstrated to be able to reduce the hepatic synthesis of apo B containing lipoprotein [48]. In addition enhanced faecal excretion of sterols has been demonstrated in several studies [49]. Furthermore the polyunsaturated fatty acids are believed to have a spatial configuration which is different to that of saturated fatty acids such that a given apolipoprotein could accommodate fewer polyunsaturated fatty acid molecules compared to saturated fatty acids. This could result in the lipoprotein bearing a lesser lipid content [50].

These mechanisms would probably explain the reduction in the total cholesterol levels seen with increased polyunsaturated fatty acid intake, which has been shown to occur mainly due to the reduction in the LDL fraction.

In our study, a reduction in LDL during the soya milk beverage feeding period was clearly seen although only marginal statistical significance could be demonstrated. Certain authorities do believe that high polyunsaturated fatty acid intake leads to a rise in the cholesterol synthesis [51]. The effect of enhanced synthesis is probably counteracted to a greater extent by the cholesterol lowering mechanisms mentioned before.

In addition to the effect of its fatty acids, soya milk may also affect the LDL levels by virtue of its protein fiber and phytosterol content. 

Many plant proteins have been implicated as affecting LDL levels. A meta-analysis by Anderson et al. [52] revealed that substituting soy protein with animal protein reduced LDL without a significant change in the HDL. Interestingly, these effects were more marked if the baseline cholesterol values were higher. In 2003 Weggemans and Trautwein [53] published a meta-analysis, which showed that when the daily intake of soy protein was 36 g/day, a dose response relation between soy intake and LDL and HDL could not be established. Thus the quantity of soy protein required to reduce the LDL is not yet established.

In an AHA review in 2006, Sacks et al. [54] point out that the earlier research which suggested that soy protein had beneficial effect on the lipids has not been confirmed. In their meta-analysis of 22 trials, the effect of protein and isoflavones derived from soya showed only very small or no benefit on the lipid parameters.

### 4.4. The Effect of SM Supplementation on the HDL Levels

The soya milk beverage supplementation in our study did not give rise to significant change in the HDL level. Although it is generally accepted that enhanced intake of PUFA reduces the HDL levels [55], this did not occur in the present study. In a study done on Sri Lankan subjects, Mendis and Kumarasunderam reported a 15% decrease in the HDL level subsequent to a dietary intervention where saturated coconut fat had been largely replaced by unsaturated soya fat [56]. The explanation probably rests with the quantum of soya fat administered. In the study of Mendis et al. the dietary soya fat content was 78 g, whereas it was only 15 g in our study. There exists evidence that a high PUFA content is associated with reduction in HDL levels. The conflicting reports which one finds in the literature regarding the effects of PUFA on HDL level can possibly be resolved by taking into account the P : S ratio in the test diets used. The HDL level has decreased consistently where the test diet contained a P : S ratio of 2.0–6.5 [57, 58]. 

 Specific unsaturated fatty acids too may have a role in the degree of reduction in the HDL fraction. Mattson and Grundy [59] found a more frequent reduction in the HDL level associated with increased linoleic acid intake compared with oleic acid. This indicates that all PUFA may not be the same in their HDL lowering effects.

In the meta-analysis by Mensink et al. [60], in which the authors analyzed 60 selected trials, the pertinent finding was that the TC/HDL ratio remained unchanged if carbohydrate replaced saturated fats, but decreased if *cis*-PUFA was the replacement. Lauric acid increased the total cholesterol mainly due to its effect on the HDL. Hence the TC/HDL ratio decreases with lauric acid rich oils.

The absence of a significant effect of the SM beverage on the HDL level in our study is probably related to the smaller quantum of polyunsaturated fat administered. 

### 4.5. Effect of Baseline LDL and HDL Levels on the Response to CM and SM  Supplementation

Many studies have indicated that free living human beings have rather precisely controlled blood cholesterol levels as increased dietary cholesterol does not elevate the serum cholesterol level in the majority of the population [61–63]. This statement however is not true when dietary fats other than cholesterol are concerned for changes in the saturation of the dietary fat intake have significant effects on the lipid parameters.

A dietary cholesterol challenge leads to the suppression of endogenous cholesterol synthesis. There may also be an associated increase in the excretion of faecal steroids and bile acids. In their study on the heterogeneity of cholesterol homeostasis in man, McNamara et al. [23] studied the response of 50 individuals comparing high versus low cholesterol diet and P  :  S*≈*1.5 diet verses P  :  S*≈*.3 diet. Amongst other parameters, they studied the response of the study subjects based on their baseline cholesterol levels. No relationship was found between the baseline cholesterol level and response to a cholesterol rich diet or change in the P  :  S of the diet.

In their discussion of the wide range seen in the HDL response to increased dietary fat, Hayek et al. make the insightful comment that it would be fruitful to investigate the correlation between the HDL response and individuals' ability to increase the flux of HDL-cholesterol ester. It would be true to say that even large studies may not be able to precisely predict the response of any given individual to dietary macronutrients if the metabolic status of the study population was extremely diverse.

In this regard Grundy et al. make the broad observation that individual responsiveness to dietary change may be influenced by genetics, environment, age, sex, or baseline LDL levels.

Hence we analyzed our data in order to determine whether the preintervention baseline levels of LDL and HDL had any effect on the subsequent response to coconut or soya milk supplementation.

As the LDL and HDL both will contribute to the total cholesterol level and because there are instances where LDL rises with concomitant lowering of HDL and vice versa, in which event the total cholesterol level would remain relatively unchanged, we approached the problem of identifying at least one important individual variability by taking the baseline of LDL and HDL separately and assessing the response in each case. Our results do indicate an important relationship, but we emphasize that further studies are required as the present study was not designed to test this particular hypothesis. The data presented must be considered as hypothesis generating requiring further work.

The decline in LDL levels reached significance only in the high baseline LDL category who received CMP and SMP supplementation. There was a clear tendency for the LDL levels to drop with CMP supplementation in the low LDL category too, although the degree of decrease did not reach statistical significance. 

On the contrary, the LDL dynamics with SMP supplementation was different. The LDL levels demonstrated a decline only in the high LDL category, whereas in the low LDL categories the LDL levels remain unchanged.

The data suggests that CMP supplementation has an action, whatever the state of the negative feedback mechanism is, and that this effect is for the better, as LDL levels consistently decline. On the other hand, SMP supplementation is effective only when the negative feedback mechanism is inefficient.

Hence CMP does not seem to cause an undesirable effect on the LDL level in any category of the study subjects and on the contrary seems to be beneficial. The study data indicates however that in the high LDL category, the introduction of SMP is as beneficial as CMP.

### 4.6. Strengths and Limitations

The main strength of the study is that it deals with practical dietetics in day-to-day living and does not concentrate on effects found solely in metabolic ward conditions. It utilized a complex food rather than extracted fat so that the multiple and diverse effects of fat, fiber, and protein on the lipid profile would be manifest. Community based dietary intervention cannot be implemented unless the advice can be put into easy practical use. Our study indicates that a traditional CM based beverage commonly used in Asian society has beneficial effects on the lipid parameters when used as a supplement.

 A limitation of the study is that it has not determined the effect of the extra caloric load in the long term. However, it would be an easy matter for dieticians to advice on the removal of a quantum of food from the normal diet to keep the energy intake within normal levels.

Reiser makes the pertinent observation that (1) without control, (2) without neutral diet, or (3) without reversing the order of oils one can draw no valid conclusions from a dietary intervention [35]. Agreeing with this statement we point out that our study was a supplementation of the normal neutral diet and that an adequate washout period was given in-between the test feeds for the study subjects to restabilize on the neutral diet again. Baseline lipids were assessed at the end of the washout period, and the results obtained confirmed that the study subjects had restabilized at their prestudy lipid level once the test feed ceased. Hence Reiser's conditions have been met for the large part in our study except the reversal of the order of administering the fats. Reversing the order of administering the fats becomes important if it is accepted that dietary intervention can reset metabolic pathways and that this change persists as metabolic memory. However, as we have clearly demonstrated that lipid levels returned to baseline at the end of the washout period, this condition cannot be deemed essential.

## 5. Conclusions

The study results indicate that CMP raises the HDL levels while reducing the LDL.

SMP significantly reduces the LDL levels only in the subjects with high baseline LDL levels. The relatively smaller quantity of soya used in this study did not appear to reduce the HDL levels.

The present study indicates that the traditional culinary methods of Asian populations using coconut milk may not lead to detrimental effects on the serum lipid profile and that changing over to PUFA need not be considered in the context of the general population. These results will have important implications for dietary advice in many countries including Sri Lanka, India, Philippines and Indonesia.

## Figures and Tables

**Table 1:** Nutritional composition of coconut milk and soya milk powder and added sago in 200 mL of porridge.

Energy and nutrient content	Coconut milk		Soya milk		Sago	
	Powder (100 g)	Porridge (36 g/200 mL)	Powder (100 g)	Porridge (52 g/200 mL)	Powder (100 g)	Porridge (4 g/200 mL)
Energy value (kJ)	2974.82	1070.93	2092	1087.84	1464.40	58.58
Protein (g)	6.80	2.45	29	15.08	0.20	0.01
Carbohydrates (g)	25.20	9.07	32.50	16.90	85.50	3.42
Fat (g)	64.80	23.33	29	15.08	0.20	0.01
Minerals (g)	1.70	0.61	4	2.08	0.10	0.00
Moisture (g)	1.50	0.54	2.50	1.30	—	—

**Table 2:** LDL and HDL levels (mg/dL) of the sample subjects before and after the consumption of coconut porridge and soya porridge.

	Coconut milk porridge (*n* = 60)			Soya milk porridge (*n* = 60)		
	Before	After	*P* value	Before	After*	*P* value
	Mean (±SD)	Mean (±SD)		Mean (±SD)	Mean (±SD)	
HDL	43.1 (±6.6)	52.7 (±13.3)	<0.01	50.4 (±9.1)	52.2 (±11.0)	0.18
LDL	133.9 (±30.1)	119.0 (±52.3)	0.02	139.3 (±47.3)	127.5 (±51.5)	0.09

*Sample size was 58.

**Table 3:** Effects of CMP and SMP supplementation on lipid parameters.

Parameter	CM	*P* value	SMP	*P* value
HDL				
Low HDL (baseline < 40.0 mg/dL)				
*n*	17		17	
Baseline	34.8 (±1.8)		45.6 (±4.8)	
Final	45.8 (±5.5)	<0.01	45.3 (±7.1)	0.86
High HDL (baseline > 40.0 mg/dL)				
*n*	43		41	
Baseline	46.4 (±4.6)		52.4 (±9.7)	
Final	55.4 (±14.4)	<0.01	55.1 (±11.1)	0.13
LDL				
Low LDL (baseline < 130.0 mg/dL)				
*n*	26		25	
Baseline	105.3 (±16.1)		113.4 (±31.3)	
Final	92.9 (±46.3)	0.42	112.3 (±48.9)	0.47
High LDL (baseline > 130.0 mg/dL)				
*n*	33		32	
Baseline	156.5 (±16.7)		158.7 (±49.3)	
Final	138.6 (±48.9)	0.05	138.8 (±52.6)	0.08

**Table 4:** Fatty acid composition of study fats.

Fatty acid	Coconut fat	Soy fat
Capric (10 : 0)	6.6	—
Lauric (12 : 0)	45.8	—
Myristic (14 : 0)	18.4	0.1
Palmitic (16 : 0)	8.0	10.7
Palmitoleic (16 : 1)	Trace	0.1
Stearic (18 : 0)	2.4	3.8
Oleic (18 : 1)	6.0	23.0
Linoleic (18 : 2)	1.7	52.4
*α*-Linolenic (18 : 3)	—	8.9
Arachidic (20 : 0)	—	0.6
Behenic (22 : 0)	—	0.4

Values given as grams fatty acids/100 grams total fat.

## Abbreviations

CM: Coconut milk
SM: Soya milk
CMP: Coconut milk porridge
SMP: Soya milk porridge
LDL: Low density lipoprotein
HDL: High density lipoprotein
PUFA: Polyunsaturated fatty acids.
